# AlgM4: A New Salt-Activated Alginate Lyase of the PL7 Family with Endolytic Activity

**DOI:** 10.3390/md16040120

**Published:** 2018-04-06

**Authors:** Guiyuan Huang, Qiaozhen Wang, Mingqian Lu, Chao Xu, Fei Li, Rongcan Zhang, Wei Liao, Shushi Huang

**Affiliations:** 1 Guangxi Key Laboratory of Marine Natural Products and Combinatorial Biosynethesis Chemistry, Guangxi Academy of Sciences, Nanning 530007, China; guiyuan1105@163.com (G.H.); wqzh-333@163.com (Q.W.); lumq520@126.com (M.L.); 18174662721@163.com (C.X.); lifei@gxas.cn (F.L.); zhangrongcan@126.com (R.Z.); laoweimail@163.com (W.L.); 2 The Food and Biotechnology, Guangxi Vocational and Technical College, Nanning 530226, China

**Keywords:** *Vibrio weizhoudaoensis*, alginate lyase, PL7 family, salt-activated enzyme

## Abstract

Alginate lyases are a group of enzymes that catalyze the depolymerization of alginates into oligosaccharides or monosaccharides. These enzymes have been widely used for a variety of purposes, such as producing bioactive oligosaccharides, controlling the rheological properties of polysaccharides, and performing structural analyses of polysaccharides. The *algM4* gene of the marine bacterium *Vibrio weizhoudaoensis* M0101 encodes an alginate lyase that belongs to the polysaccharide lyase family 7 (PL7). In this study, the kinetic constants *V*_max_ (maximum reaction rate) and *K*_m_ (Michaelis constant) of AlgM4 activity were determined as 2.75 nmol/s and 2.72 mg/mL, respectively. The optimum temperature for AlgM4 activity was 30 °C, and at 70 °C, AlgM4 activity dropped to 11% of the maximum observed activity. The optimum pH for AlgM4 activity was 8.5, and AlgM4 was completely inactive at pH 11. The addition of 1 mol/L NaCl resulted in a more than sevenfold increase in the relative activity of AlgM4. The secondary structure of AlgM4 was altered in the presence of NaCl, which caused the α-helical content to decrease from 12.4 to 10.8% and the β-sheet content to decrease by 1.7%. In addition, NaCl enhanced the thermal stability of AlgM4 and increased the midpoint of thermal denaturation (Tm) by 4.9 °C. AlgM4 exhibited an ability to degrade sodium alginate, poly-mannuronic acid (polyM), and poly-guluronic acid (polyG), resulting in the production of oligosaccharides with a degree of polymerization (DP) of 2–9. AlgM4 possessed broader substrate, indicating that it is a bifunctional alginate lyase. Thus, AlgM4 is a novel salt-activated and bifunctional alginate lyase of the PL7 family with endolytic activity.

## 1. Introduction

Alginic acid has the ability to form viscous solutions and gels in aqueous media and is nontoxic to living organisms. Therefore, it has been widely used in the pharmaceutical, cosmetic, food, and biotech industries [1]. In addition, the degradation products of alginic acid—alginate oligosaccharides—have a wide range of biological activities, such as the promotion of growth and the alleviation of abiotic stress in plants; antitumour, antibacterial, anti-inflammatory, anticoagulant, antioxidative, and immunomodulatory activities; and the reduction of free radicals and blood glucose and lipids. Alginate oligosaccharides have broad application prospects in the green agriculture, medical, food, and household chemical industries, among others [2].

Alginic acid, also known as algin or alginate, is a straight-chain polysaccharide composed of β-d-mannuronic acid (M) and its C5 stereoisomer α-l-guluronic acid (G), which are randomly linked via α-1,4-glycosidic bonds. Alginate molecules can have the sugar monomers M and G arranged in three ways: M monomers can be linked in succession, forming poly-mannuronic acid (polyM); G monomers can be linked in succession, forming poly-guluronic acid (polyG); and M and G monomers can be randomly and alternately linked, forming poly-guluronic acid -mannuronic acid (polyMG) [3]. The relative proportions of M and G vary among the alginic acids derived from different organisms [4,5].

Alginate lyases catalyze the cleavage of the 1,4-glycosidic bonds between the uronic acid monomers in alginate, resulting in the production of oligouronic acids or uronic acid monomers. Alginate lyases are present in a wide range of organisms and can be isolated from marine algae, molluscs, and microorganisms, as well as soil microorganisms. Currently, the primary sources of alginate lyases are marine bacteria, including members of the genera *Pseudomonas* and *Vibrio* [6].

The catalytic mechanisms of alginate lyases are well understood. Alginate lyases catalyze the cleavage of 1,4-glycosidic bonds in alginic acid via a β-elimination reaction. In addition, a double bond is formed between C4 and C5 of the saccharide ring containing the 4-*O* glycosidic bond, generating an oligomer with 4-deoxy-l-erythro-hex-4-enepyranosyluronate at the nonreducing end [7]. Based on their catalytic characteristics, alginate lyases are divided into endolytic and exolytic alginate lyases [8,9]. According to the substrate specificity, endolytic alginate lyases can be divided into mannuronate lyases (polyM lyase, EC 4.2.2.3) and guluronate lyases (polyG lyase, EC 4.2.2.11). Bifunctional enzymes that exhibit activities towards both polyG and polyM have also been identified [10]. While numerous alginate lyases have been discovered, few studies have focused on the enzymatic properties of alginate lyases. In recent years, a number of alginate lyases have been discovered and reported, including the cold-adapted alginate lyases [11,12,13], thermostable alginate lyases [14], high-alkaline alginate lyases [15], and salt-activated alginate lyases [12,15,16,17]. According to the evolution and homology of amino acid sequences, most alginate lyases belong to seven families of polysaccharide lyases (PL-5, PL-7, PL-14, PL-15, PL-17, and PL-18) [18]. Most of the reported alginate lyases have endolytic activity [15,19,20,21] and hydrolyze sodium alginate (SA) to produce oligosaccharides. Alginate lyase A1-IV, produced by the bacterial strain *Sphingomonas* sp. A1 [22], and alginate lyase Atu3025, produced by *Agrobacterium tumefaciens* C58 [23], possess exolytic activity and hydrolyze SA into mannuronate or guluronate. Alginate lyases that degrade alginate into monosaccharides are part of the PL15 family [22,23], whereas alginate lyases with endolytic activity belong to the PL-5, 6, 7, 14, 16, 17, and 18 families [24].

The marine bacterium *Vibrio weizhoudaoensis* M0101 harbors the *algM4* gene, which encodes an alginate lyase belonging to polysaccharide lyase family 7 (PL7). In this study, we purified exogenously expressed AlgM4 and observed it to exhibit high salt tolerance, as AlgM4 activity increased more than sevenfold in the presence of 1 mol/L NaCl. The result is different from those for other salt-activated alginate lyases for which enzyme activity is decreased at 1 mol/L NaCl [15,25]. In the depolymerization of a high content of sodium alginate, Alg2A generated equal total molar amounts of oligosaccharides, but the amounts of oligosaccharides with DP of 5–10 were higher than those for both of the two commercial enzymes [26]. AlgM4 showed activities toward both polyM and polyG, which may degrade alginate more effectively. Moreover, AlgM4 catalyzing polyM released oligosaccharides with DP 7–9 from the polyM, which was different from the previously reported endolytic alginate lyases, despite their diverse substrate specificities [27,28]. Therefore, the unique endolytic reaction mode of AlgM4 gives it a distinct advantage in facilitating uronic acid oligosaccharides with high DPs. AlgM4 could be a good tool for the preparation of alginate oligosaccharides. AlgM4 not only functions as a key enzyme in the preparation and functional study of oligosaccharides but also plays an important role in utilization of alginate for ethanol fermentation.

## 2. Results and Discussion

### 2.1. Analysis of the AlgM4 Sequence

**Figure 1:**
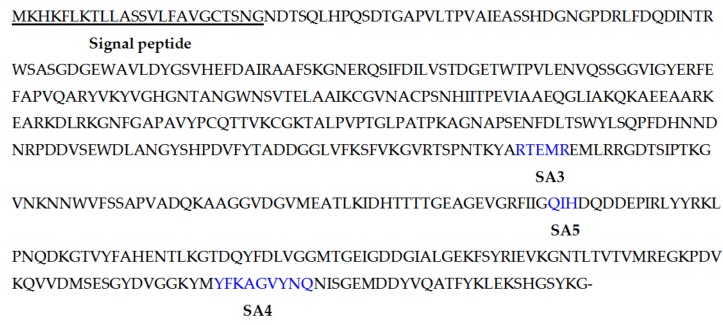
The deduced amino acid sequences of AlgM4. The signal peptide is underlined; SA3, SA5, and SA4 are indicated with blue symbols.

**Figure 2:**
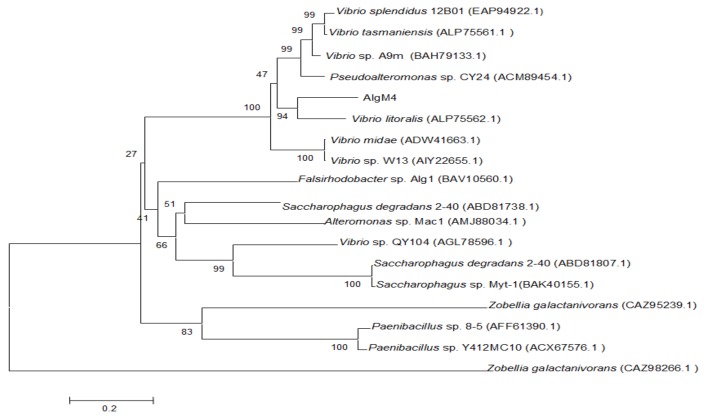
Neighbor-joining phylogenetic tree of *V. weizhoudaoensis* strain M0101 based on putative AlgM4 protein sequences.

The alginate lyase gene *algM4* is 1563 bp in length and encodes a 520-amino-acid protein. A signal peptide analysis of AlgM4 using SignalP 4.0 (http://www.cbs.dtu.dk/services/SignalP/) predicted that the N-terminus of AlgM4 contains a 24-amino-acid signal peptide (Figure 1). A sequence alignment using the National Center for Biotechnology Information (NCBI) BLAST (https://blast.ncbi.nlm.nih.gov/) engine revealed that AlgM4 is a dual-domain protease, containing an N-terminal F5/8 type C domain and a C-terminal alginate-lyase 2 domain. The alginate lyases of the PL7 family contain three highly conserved domains: SA3 (RXEXR), SA4 (YXKAGXYXQ), and SA5 (QXH). The BLAST sequence analysis showed that AlgM4 contains the conserved amino acid sequences of the SA3 domain (RTEMR), the SA4 domain (YFKAGVYNQ), and the SA5 catalytic domain (QIH) (Figure 1). Therefore, AlgM4 belongs to the PL7 family of alginate lyases. The amino acid sequence of AlgM4 was compared with the sequences of other alginate lyases of the PL7 family selected in the CAZy database (http://www.cazy.org/), and a phylogenetic tree was constructed using the neighbor joining method (Figure 2). AlgM4 was most closely related to an alginate lyase from *Vibrio litoralis* BZM-2 (ALP75562.1), with an amino acid sequence similarity of 74% observed between AlgM4 and ALP75562.1, without annotation by genome analysis. These results suggest that AlgM4 is a new alginate lyase of the PL7 family.

### 2.2. Purification and Enzymatic Activity of AlgM4

**Figure 3:**
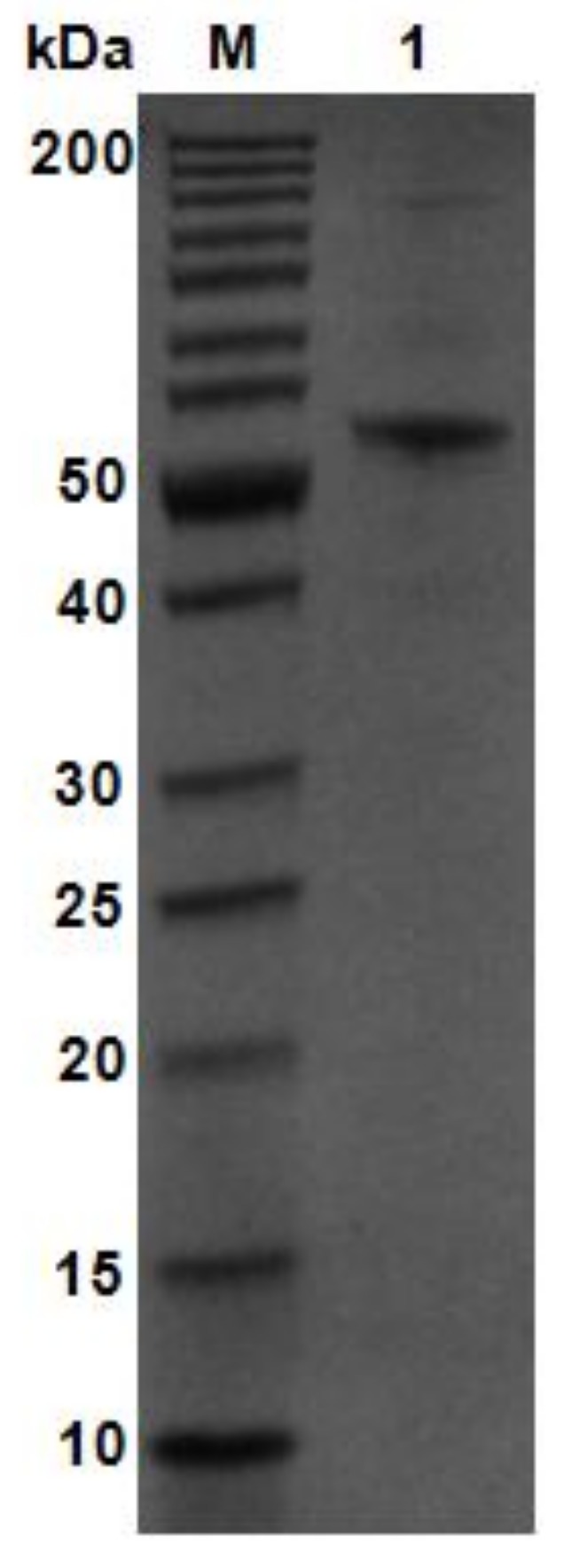
SDS-PAGE analysis of purified AlgM4. Lane M, molecular weight markers; Lane 1, purified AlgM4.

Using *V. weizhoudaoensis* M0101 genomic DNA as a template, the *algM4* gene was PCR amplified without the N-terminal signal peptide sequence or the stop codon and ligated into the pET30a(+) plasmid. The plasmid was then transformed into *Escherichia coli* BL21 (DE3) cells for AlgM4 expression. Purified AlgM4 protein with a C-terminal 6×histidine (His) tag was obtained by Ni^2+^ affinity chromatography. Sodium dodecyl sulfate polyacrylamide gel electrophoresis (SDS-PAGE) showed a single protein band with a molecular weight of approximately 55 kDa (Figure 3). The specific activity of the purified AlgM4 was 4638 U/mg (Table 1), exhibiting a 36.7% increase in specific activity compared with the crude enzyme extract.

### 2.3. V_max_ (Maximum Reaction Rate) and K_m_ (Michaelis Constant) of AlgM4

AlgM4 activity was measured when incubated with various concentrations of substrate (SA) in a water bath at 30 °C for 10 min, and the resulting values were used to calculate the kinetic constants of AlgM4 activity. The *V*_max_ and *K*_cat_ values of AlgM4 were 2.75 nmol/s and 30.25 S^−1^, respectively, and the *K*_m_ value was 2.72 mg/mL. Two other NaCl-activated enzymes—AlyPM from *Pseudoalteromonas* sp. SM0524 and AlySY08 from *Nitratiruptor* sp. SB155-2—were previously shown to have *K*_m_ values of 74.39 [17] and 0.36 mg/mL [14], respectively. The oligoalginate lyase Alg17C derived from *Saccharophagus degradans* 2-40 had an observed *K*_m_ value of 35.2 mg/mL [18]. *Vibrio splendidus* 12B01 expresses three oligoalginate lyases—OalA, OalB, and OalC—which had observed *K*_m_ values of 3.25, 0.76, and 0.53 mg/mL, respectively [29].

### 2.4. Enzymatic Characteristics of AlgM4

**Figure 4:**
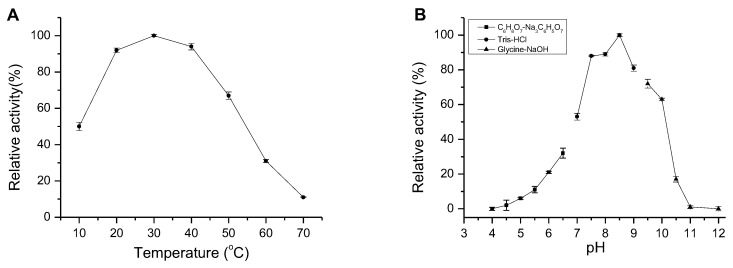
Effect of temperature and pH on AlgM4 activity. (**A**) The optimal temperature for AlgM4 activity. The activity of AlgM4 at 30 °C was completely retained; (**B**) The optimal pH for AlgM4 activity. The activity of AlgM4 at pH 8.5 was completely retained.

AlgM4 exhibited high enzymatic activity at 20–40 °C, which decreased to 11% of the maximal observed activity when incubated at 70 °C. Similar to other NaCl-activated alginate lyases in the PL7 family (such as A1m [15], rA9mT [16], and AlyPM [17]), AlgM4 had highest enzymatic activity at 30 °C (Figure 4A).

Many alginate lyases have optimal pH values between pH 7 and 8, in contrast to the optimal high-alkaline pH values of pectate lyases [20]. The optimum pH for AlgM4 activity was assessed, and the results are shown in Figure 4B. The optimum reaction pH was 8.5, indicating that AlgM4 was weakly basophilic. In slightly alkaline environments (pH 7.5–9.0), AlgM4 retained 80% activity, whereas AlgM4 was completely inactive at pH 11.

**Figure 5:**
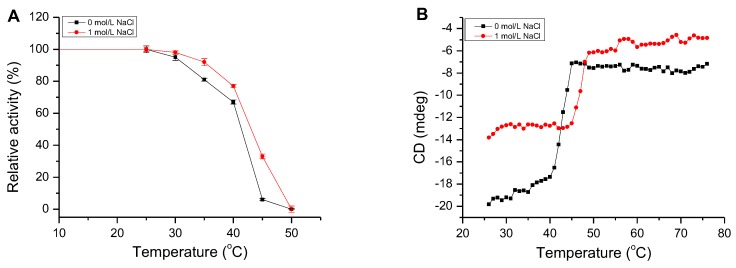
The thermal stability and melting temperature (Tm) of AlgM4. (**A**) The thermal stability of AlgM4. The residual activity of AlgM4 at 25 °C was completely retained; (**B**) Circular dichroism signals at 218 nm were used for analysis of the Tm value.

The activity of AlgM4 remained stable for 30 min at 25 °C, regardless of the presence or absence of 1 mol/L NaCl (Figure 5A). After a 30 min incubation at 30 or 35 °C in the presence of 1 mol/L NaCl, AlgM4 retained 92% of its initial activity, which decreased rapidly at temperatures exceeding 40 °C and decreased by 63% at 45 °C. In the absence of NaCl, the enzymatic activity of AlgM4 was reduced by 94% at 45 °C. An examination of temperature tolerance showed that NaCl improved the thermal stability of AlgM4.

The effects of metal ions and surfactants on AlgM4 activity are shown in Table 2. Ca^2+^ did not influence enzyme activity, unlike for other alginate lyases [12,17,20]. While Mg^2+^ promoted the activity of AlgM4, Cu^2+^, Mn^2+^, and Zn^2+^ inhibited AlgM4 activity. The most significant inhibitory effect on AlgM4 was observed by Zn^2+^, which caused an 82% reduction in AlgM4 activity. Ethylene diamine tetraacetic acid (EDTA) and SDS suppressed AlgM4 activity to varying degrees. The anionic surfactant SDS strongly inhibited the enzymatic activity of AlgM4, causing a 97% reduction in AlgM4 activity, while EDTA reduced AlgM4 activity by 35%.

### 2.5. Effect of NaCl on AlgM4 Activity

Different concentrations of NaCl were added to 0.1 mg/mL purified AlgM4, and the enzymatic activity of AlgM4 was then examined. AlgM4 was promoted at NaCl concentrations of 0.1–1.4 mol/L and was greatest in the presence of 1.0 mol/L NaCl, exhibiting more than 7 times the activity observed in the absence of NaCl (Table 3). The activity of AlyPM in the presence of 0.5–1.2 mol/L NaCl was 6 times that observed in the absence of NaCl [17]. Under optimum reaction conditions, the activity of A1m in the presence of 0.6–0.8 mol/L NaCl was 20 times that observed in the absence of NaCl [15]. The activity of rA9mT in the presence of 0.4 mol/L NaCl was 24 times that observed in the absence of NaCl [16].

### 2.6. Determination of the Secondary Structure and Thermal Denaturation Temperature of AlgM4

The thermal denaturation temperature of AlgM4 was determined by circular dichroism (CD) spectroscopy at 25–75 °C. In addition, the secondary structure of AlgM4 was determined by ultraviolet–visible (UV–Vis) spectroscopy and CD spectroscopy. The CD absorption values of AlgM4 protein at 218 nm in different temperatures are shown in Figure 5B, and the CD values were used for analysis of the Tm value. The CD values of AlgM4 relatively remained stable at 25–35 °C in the absence of NaCl; subsequently, CD values slowly increased with the increase of temperature and the denaturation of AlgM4, then increased dramatically at 40–45 °C and remained stable at temperatures exceeding 45 °C. The AlgM4 protein was denatured completely when the temperature was higher than 45 °C. However, in the presence of 1 mol/L NaCl, the CD values increased rapidly at 45–50 °C and remained stable at temperatures exceeding 50 °C. The results showed that NaCl enhanced the ability of AlgM4 to resist thermal denaturation.

**Figure 6:**
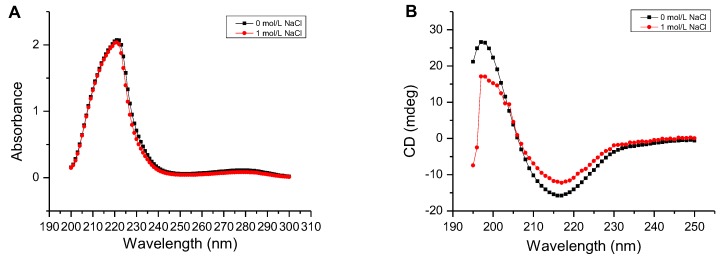
Determination of the secondary structure of the AlgM4. (**A**) Determination of the secondary structure by UV–Vis absorption spectra; (**B**) Determination of the secondary structure by circular dichroism.

As shown in Figure 6A, the UV absorbance of AlgM4 at 220–240 nm was significantly reduced in the presence of 1 mol /L NaCl. CD spectroscopy was used to determine the secondary structure of the purified AlgM4, and the results are shown in Figure 6B. The absorption spectrum of AlgM4 was characterized by positive and negative peaks at approximately 198 and 218 nm, respectively, and the intensity of these peaks was reduced by the addition of 1 mol/L NaCl. The results showed that the α-helix and β-sheet contents in the secondary structure of AlgM4 were decreased after addition of 1 mol/L NaCl. Specifically, the α-helix and β-sheet contents were reduced from 12.4% and 38.2% to 10.8% and 36.5%, respectively. The changes in the contents of secondary structural elements are summarized in Table 4. The secondary structure of AlgM4 was altered in the presence of 1 mol/L NaCl, which may enhance the affinity of the enzyme for its substrates and facilitate enzymolysis. The alginate lyase AlyPM, derived from *Pseudoalteromonas* sp. SM0524, differs from AlgM4 in that the presence of NaCl did not alter the secondary structure of AlyPM. However, NaCl enhanced the affinity of AlyPM for its substrates, thereby promoting enzymolysis [17]. In addition, AlyPM is a cold-adapted enzyme, and its thermal denaturation temperature is relatively low, with a Tm of 37 °C [17]. At 1 mol/L, NaCl not only altered the secondary structure of AlgM4 but also enhanced the ability of AlgM4 to resist thermal denaturation. As a result, the midpoint of thermal denaturation (Tm) was increased from 43.3 to 48.2 °C (Figure 5B).

### 2.7. Analysis of the Products of AlgM4-Mediated Enzymolysis of Alginate by Ultra-Performance Liquid Chromatography (UPLC)–Quadrupole Time-of-Flight (QTOF)–Mass Spectrometry (MS)/MS

**Figure 7:**
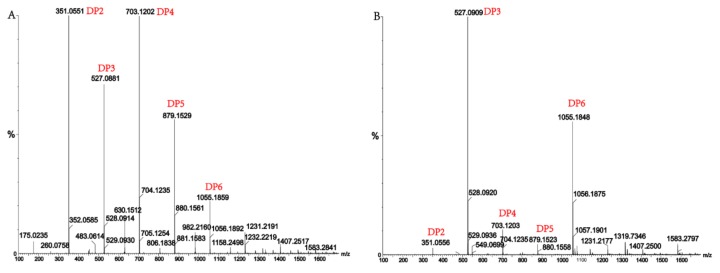
(**A**) Ultra-Performance Liquid Chromatography (UPLC)–Quadrupole Time-of-Flight (QTOF)–MS/MS analysis of hydrolysates of AlgM4 with sodium alginate as the substrate; (**B**) UPLC–QTOF–MS/MS analysis of hydrolysates of AlgM4 with polyG as the substrate. DP indicates the degree of polymerization of oligosaccharides from the alginate lyase hydrolysates.

**Figure 8:**
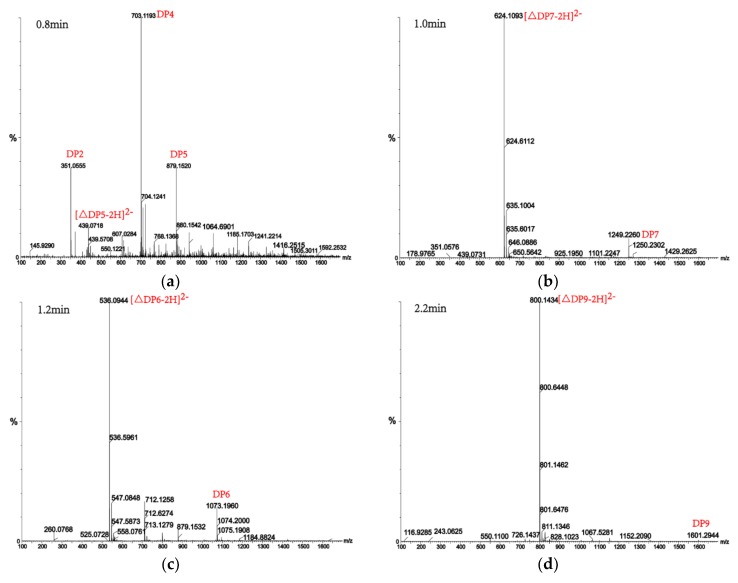

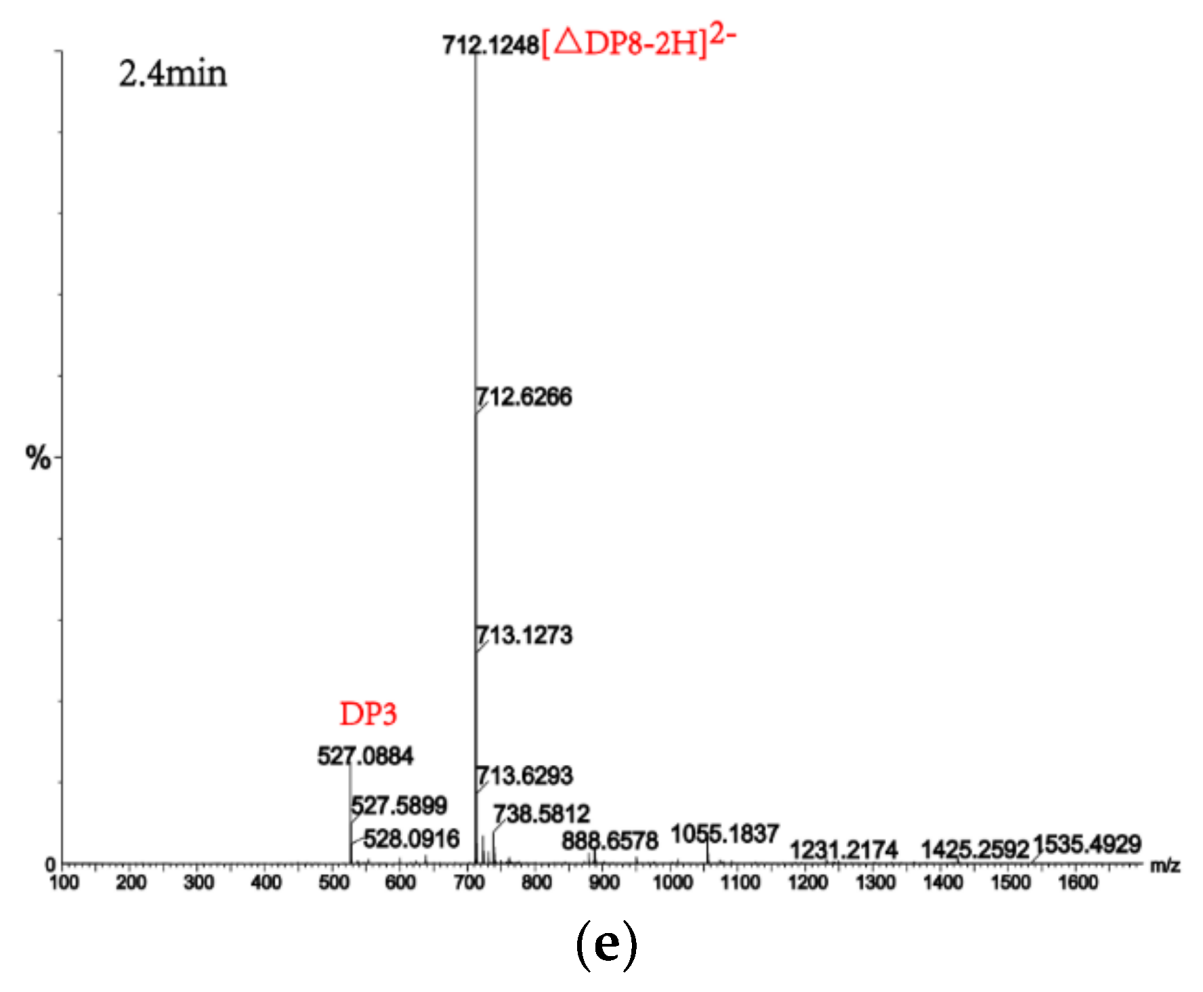
UPLC–QTOF–MS/MS analysis of hydrolysates of AlgM4 with polyM as the substrate. DP indicates the degree of polymerization of oligosaccharides from the alginate lyase hydrolysates. The reaction products of disaccharide, tetrasaccharide and pentasaccharide were detected at 0.8 min (**a**). The major peacks of DP7, DP6, DP9 and DP8 were detected at 1.0 min (**b**), 1.2 min (**c**), 2.2 min (**d**) and 2.4 min (**e**), respectively.

Alginate lyases with endolytic characteristics generally act on glycosidic bonds within the linear polysaccharide chain of alginate, generating unsaturated oligosaccharides that are dominated by disaccharides, trisaccharides, and tetrasaccharides [27]. Exolyases further depolymerize these oligosaccharides into mannuronic acids [18,29,30]. AlgM4 degrades both SA (Figure 7A) and polyG (Figure 7B) to produce oligosaccharides with degree of polymerization DP 2–6 [31,32]. The content of each oligosaccharide can be determined only after quantitative determination. Unlike SA and polyG, degradation of polyM produces oligosaccharides DP7, DP8, and DP9 (Figure 8). Furthermore, AlgM4 may be useful in the preparation of oligosaccharides, especially with high DP 7–9, and the study of their biological functions.

In recent years, the biological activities of oligosaccharides and the application of oligosaccharides in the medical and biotechnology fields has attracted the attention of researchers [33]. Oligosaccharides have higher degrees of polymerization and possibly better bioactivity [26]. An et al. observed that oligosaccharides (DP 6–8) derived from SA stimulated the accumulation of phytoalexin and induced phenylalanine ammonia lyase in soybean cotyledons, resulting in their acquired resistance to *Pseudomonas aeruginosa* [34]. In addition, trisaccharides, tetrasaccharides, pentasaccharides, and hexasaccharides obtained by enzymatic degradation of SA promoted the growth of lettuce seedlings [35].

## 3. Materials and Methods

### 3.1. The Bacterium

The marine bacterium *V. weizhoudaoensis* M0101 was isolated from rotten Sargassum collected from Weizhou Island, Beihai, Guangxi Province, China.

### 3.2. Cloning and Expression of the algM4 Gene

The primers were designed according to the nucleic acid sequence of the *algM4* gene. The following primers were used to amplify the *algM4* gene: upstream primer, 5′-GGAATTCCATATGCTTGCATCTTCTGTG-3′ (the *Nde*I restriction site is underlined); downstream primer, 5′-CCGCTCGAGACCTTTATAAGAACCGTG-3′ (the *Xho*I restriction site is underlined). The parameters of the polymerase chain reaction (PCR) were as follows: 94 °C for 2 min; followed by 30 cycles of 94 °C for 30 s, 58 °C for 30 s, and 72 °C for 1 min 40 s; with a final incubation at 72 °C for 10 min. The PCR product was double digested with the *Nde*I and *Xho*I restriction enzymes and then ligated into the pET30a(+) vector to construct the recombinant plasmid pET30a-*algM4*. To induce the expression of AlgM4, the recombinant plasmid was transformed into the *E. coli* strain BL21 (DE3). The positive transformants were picked, inoculated into 10 mL of LB (Luria-Bertani) medium containing 50 μg/mL kanamycin (Kan) and cultured at 37 °C with shaking (200 rpm) until the OD600 of the culture reached 0.5. The culture was then inoculated into LB medium containing 50 μg/mL Kan (inoculum volume: 1% (*v*/*v*)) and cultivated until the OD600 value reached 0.5. Subsequently, isopropyl β-d-1-thiogalactopyranoside (IPTG) was added at a final concentration of 0.5 mmol/L. After the addition of IPTG, the bacteria were cultured for another 12 h at 25 °C with shaking (200 rpm).

### 3.3. Purification of AlgM4

Protein isolation and purification was carried out at 0–4 °C. The IPTG-induced bacteria were harvested by centrifugation at 6000 rpm for 10 min. Subsequently, the bacteria were resuspended in buffer (10 mmol/L imidazole, 300 mmol/L NaCl, and 20 mmol/L Tris-HCl; pH 7.0) and lysed by ultrasonication. The lysates were centrifuged at 12,000 rpm for 30 min, and the resulting crude enzyme solution was collected. After washing off the protein impurities with buffer (50 mmol/L imidazole, 300 mmol/L NaCl, and 20 mmol/L Tris-HCl; pH 7.0), AlgM4 bound to the Ni^2+^ column was eluted with elution buffer containing 150 mmol/L imidazole, 300 mmol/L NaCl, and 20 mmol/L Tris-HCl (pH 7.0). The purified protein was analyzed by SDS-PAGE, and the protein concentration was determined using the Bradford method.

### 3.4. Determination of the Enzymatic Properties of AlgM4

Enzymatic properties were determined using 0.1 mg/mL AlgM4. Nine hundred microliters of a 1.0% (*w*/*v*) SA solution was mixed with 100 μL of purified AlgM4 and incubated in a water bath at 30 °C for 10 min. The mixture was then boiled in a water bath for 5 min to terminate the reaction. After the reaction system was cooled to room temperature, the absorbance was measured at 235 nm. The unit of enzymatic activity (U) was defined as an increase in absorbance of 0.01 per minute. To determine the optimum reaction temperature, the enzymatic activity of AlgM4 was measured at 10–70 °C in 100 mmol/L Tris-HCl (pH 7.0) buffer using 0.2% (*w*/*v*) SA as a substrate. To determine the optimum reaction pH, the activity of AlgM4 was evaluated in 100 mmol/L citric acid–sodium citrate buffer (pH value: 4.5–6.5), 100 mmol/L Tris-HCl buffer (pH value: 7.0–9.0), or 100 mmol/L glycine-sodium hydroxide buffer (pH value: 9.5–12.0) at the optimum reaction temperature. To test the thermal stability of the enzyme, AlgM4 was first incubated at 25–50 °C for 30 min, and the residual AlgM4 activity was then measured under the optimum reaction conditions. To examine the effects of metal ions and surfactants on the enzymatic activity of AlgM4, metal ions (Ca^2+^, K^+^, Mg^2+^, Mn^2+^, Zn^2+^, and Cu^2+^), EDTA, or SDS were added to 50 μL of enzyme solution at a final concentration of 1 mmol/L. The activity of AlgM4 was then measured under the optimum reaction conditions using a 0.2% (*w*/*v*) SA solution as a substrate. The effect of NaCl on AlgM4 activity was also examined. NaCl was added to the enzyme solution at final concentrations of 0.1–1.4 mol/L, and the activity of AlgM4 was then compared to that observed under optimum reaction conditions.

### 3.5. Determination of the Kinetic Constants for AlgM4 Activity

The enzymatic activity of AlgM4 (0.1 mg/mL) was measured under the optimum reaction conditions using 3,5-dinitrosalicylic acid (DNS) assay. The concentrations of the substrate, SA, assayed were 0.5–10 mg/mL. Lineweaver-Burk (double-reciprocal) plots were generated using 1/[*S*] as the abscissa and 1/*V* as the ordinate. The *V*_max_ and *K*_m_ values were calculated using the Michaelis-Menten equation.

### 3.6. The Effects of NaCl on the Secondary Structure and Thermal Stability of AlgM4

In one group, NaCl was added to a 300 μL reaction containing 0.28 mg/mL of purified AlgM4 (final concentration of NaCl: 1 mol/L), while another group was not exposed to NaCl. The secondary structure of the purified AlgM4 protein was determined at 25 °C using a TU-1901 Dual Beam Ultraviolet Spectrophotometer (PERSEE, Beijing, China) (spectral range: 200–350 nm) and a Chirascan Circular Dichroism Spectrometer (Applied Photophysics Ltd., Surrey, UK) (spectral range: 195–250 nm; optical path length: 10 mm; bandwidth: 0.5 nm). The composition of the secondary structural elements was analyzed using CDpro software (http://sites.bmb.colostate.edu/sreeram/CDPro/CDPro.htm). The thermal denaturation temperature of AlgM4 was measured under the following conditions: spectral range, 200–260 nm; bandwidth, 0.7 nm; 25–75 °C. The Tm value was calculated using Global 3 software (Applied Photophysics Ltd., Surrey, UK).

### 3.7. Analysis of the Products of AlgM4-Mediated Enzymolysis Using UPLC–QTOF–MS/MS

The purified AlgM4 (0.25 mg/mL) enzyme was mixed with an equal volume of 1.0% (*w*/*v*) SA, polyM, or polyG; incubated in a water bath at 30 °C for 6 h; and then concentrated in vacuo. After high-speed centrifugation, the supernatants were collected and filtered through filter membranes with a 0.22 µm pore size. The filtrates were analyzed using liquid chromatography (LC)-MS. The equipment used in the analysis was a UPLC-QTOF-MS/MS system (Waters Corporation, Milford, MA, USA), which consisted of a UPLC I-Class instrument (Waters Corporation, Singapore) and a XEVO G2-S mass spectrometer (Waters Corporation, Milford, MA, USA). The operating conditions of LC were as follows: ACQUITY UPLC HSS T3 C18 column (2.1 mm × 100 mm, 1.8 μm, Waters Corporation, Milford, MA, USA); gradient elution with 0.1% formic acid-water (A) and formic acid-acetonitrile (B); flow rate, 0.5 mL/min; column temperature, 35 °C; analytic time, 10 min; injection volume, 1.0 μL. The MS conditions were as follows: ion scan mode, negative-ion ESI mode; scan range, 100–1700 Da; ion source temperature, 100 °C; desolvation gas temperature, 400 °C; desolvation gas flow rate, 1000 L/h; capillary voltage, 2.5 kV; cone voltage, 40 V; low collision energy, 6 V; high collision energy, 35–50 V; data acquisition software, MassLynx 4.1 SCN 884 (Waters Corporation, Milford, MA, USA); data acquisition mode, MSE.

## 4. Conclusions

AlgM4 is a new salt-activated and bifunctional alginate lyase of the PL7 family with endolytic activity derived from the marine bacterium *V*. *weizhoudaoensis* M0101. Compared with the observed AlgM4 activity in the absence of NaCl, the enzymatic activity of AlgM4 increased in the presence of various concentrations of NaCl (0.1–1.4 mol/L). The addition of 1 mol/L NaCl resulted in a more than sevenfold increase in AlgM4 activity. Therefore, AlgM4 tolerates high-salinity environments. NaCl not only altered the composition of the secondary structural elements in AlgM4 but also enhanced its thermal stability. AlgM4 hydrolyzed SA, polyM, and polyG via its endolytic activity, producing oligosaccharides (DP 2–9). The alginate lyase AlgM4 has an important application value in the preparation of bioactive oligosaccharides and in the processes of alginate saccharification and ethanol fermentation.

## Figures and Tables

**Table 1:** Summary of AlgM4 purification.

Steps	Total Protein (mg)	Total Activity (U)	Specific Activity (U/mg)	Recover (%)	Purification (Fold)
Crude enzyme AlgM4	58.352	73878	1266	100	1
Purified AlgM4	11.55	53570	4638	72.5	3.7

**Table 2:** Effect of chemical reagents on AlgM4 activity.

Reagent	Concentration (mmol/L)	Relative Activity (%)
None	-	100 ± 0.3
CaCl_2_	1	97 ± 0.1
MgCl_2_	1	114 ± 1.1
KCl	1	91 ± 0.9
CuCl_2_	1	75 ± 1.7
MnCl_2_	1	77 ± 2.4
ZnCl_2_	1	18 ± 4.5
EDTA	1	65 ± 6.2
SDS	1	3 ± 3.1

The data are expressed as the means ± SD, *n* = 3. The activity of AlgM4 in the absence of chemical reagents was completely retained.

**Table 3:** Effect of NaCl on AlgM4 activity.

NaCl Concentration (mol/L)	Relative Activity (%)
0	100 ± 0.2
0.1	229 ± 5.6
0.2	286 ± 6.4
0.3	356 ± 3.8
0.4	447 ± 1.4
0.5	481 ± 0.8
0.6	528 ± 3.1
0.7	572 ± 0.4
0.8	628 ± 3.4
0.9	664 ± 1.9
1.0	741 ± 3.2
1.2	462 ± 5.8
1.4	236 ± 4.5

The data are expressed as the means ± SD, *n* = 3. The activity of AlgM4 in the absence of NaCl was completely retained.

**Table 4:** Secondary structure of AlgM4 as estimated by CD.

Enzyme	α-Helix (%)	β-Sheet (%)	β-Turu (%)	Random Coil (%)
AlgM4	12.4	38.2	21.3	28
AlgM4 + 1 mol/L NaCl	10.8	36.5	22.9	29.6
